# Primary care physicians’ perceptions of artificial intelligence systems in the care of adolescents’ mental health

**DOI:** 10.1186/s12875-024-02417-1

**Published:** 2024-06-13

**Authors:** Pooria Ghadiri, Mark J. Yaffe, Alayne Mary Adams, Samira Abbasgholizadeh-Rahimi

**Affiliations:** 1 Department of Family Medicine and Faculty of Dental Medicine and Oral Health Sciences, McGill University, 5858 Ch. de la Côte-des-Neiges, Montréal, QC H3S 1Z1 Canada; 2 Mila-Quebec AI Institute, Montréal, QC Canada; 3 Lady Davis Institute for Medical Research (LDI), Jewish General Hospital, Montréal, QC Canada; 4 St. Mary’s Hospital Center of the Integrated University Centre for Health and Social Services of West Island of Montreal, Montréal, QC Canada

**Keywords:** Adolescent health, Mental health, Artificial intelligence, Innovative technologies, Primary care physicians, Family physicians, Pediatricians

## Abstract

**Background:**

Given that mental health problems in adolescence may have lifelong impacts, the role of primary care physicians (PCPs) in identifying and managing these issues is important. Artificial Intelligence (AI) may offer solutions to the current challenges involved in mental health care. We therefore explored PCPs’ challenges in addressing adolescents’ mental health, along with their attitudes towards using AI to assist them in their tasks.

**Methods:**

We used purposeful sampling to recruit PCPs for a virtual Focus Group (FG). The virtual FG lasted 75 minutes and was moderated by two facilitators. A life transcription was produced by an online meeting software. Transcribed data was cleaned, followed by a priori and inductive coding and thematic analysis.

**Results:**

We reached out to 35 potential participants via email. Seven agreed to participate, and ultimately four took part in the FG. PCPs perceived that AI systems have the potential to be cost-effective, credible, and useful in collecting large amounts of patients’ data, and relatively credible. They envisioned AI assisting with tasks such as diagnoses and establishing treatment plans. However, they feared that reliance on AI might result in a loss of clinical competency. PCPs wanted AI systems to be user-friendly, and they were willing to assist in achieving this goal if it was within their scope of practice and they were compensated for their contribution. They stressed a need for regulatory bodies to deal with medicolegal and ethical aspects of AI and clear guidelines to reduce or eliminate the potential of patient harm.

**Conclusion:**

This study provides the groundwork for assessing PCPs’ perceptions of AI systems’ features and characteristics, potential applications, possible negative aspects, and requirements for using them. A future study of adolescents’ perspectives on integrating AI into mental healthcare might contribute a fuller understanding of the potential of AI for this population.

**Supplementary Information:**

The online version contains supplementary material available at 10.1186/s12875-024-02417-1.

## Introduction

### Adolescents and mental health

Adolescence is a transitional stage of physical, psychological, social and moral development between childhood and adolescents, defined by the World Health Organization as the between the ages 10 and 19 [1, 2]. The search for greater independence and self-reliance commonly experienced during these years may be challenging for those with emotional or social concerns. These challenges can provoke the development or worsening of mental health problems in adolescents, and negatively impact the lives of affected individuals, their families, and their communities [3–5]. Anxiety, depression, and substance misuse disorders may arise and cause adolescents to struggle with self-regulation and impulse control [6, 7].

In Canada, one in five persons experiences a mental health problem [8] and about half of those who have been identified in adulthood as having a mental health condition began experiencing related symptoms before the age of 14 [9]. Between ages 17 and 19, one in four suffers from depression or anxiety, with half of them having attempted suicide or engaged in self-harm [10]. In the U.K. the number of non-suicidal self-harming behaviours has almost quadrupled over the last decade, while suicide has nearly doubled for every 100,000 adolescents [11, 12]. Worldwide, approximately 140,000 people aged 10 to 24 succumb to suicide annually [13], and in the USA and Canada, suicide is the second leading cause of mortality [14, 15]. The Canadian Institute for Health Information has reported that adolescents’ emergency department visits for mental health problems climbed by 61% between 2009 and 2019 [8]. The main contributor to disability adjusted life years lost by adolescents is depression, resulting in high social and economic cost over the life course [16]. Optimizing ‘gateways into primary care’ is crucial for addressing the adolescents’ mental health crisis by providing timely interventions aimed at prompt and adequate treatment [16]. This may bridge the gap between recognition and care, fostering a healthier, more resilient future generation [16].

### Adolescents and primary care

Primary care (PC) has been defined as “the provision of integrated, accessible healthcare services by clinicians who are accountable for addressing a large majority of personal healthcare needs, developing a sustained partnership with patients, and practicing in the context of family and community” [17]. Best practice standards for adolescents promote involvement of primary care physicians (PCPs) [18, 19] doing physical exams, screening for risky behaviors, and attempting to build trusting relationships [20].

Delivery of such care may be problematic. Adolescents visit PCPs far less than the general population [20, 21]; and when they do, they may be “reluctant historians” [22] and/or lack trust in PCPs or the services they deliver [23]. As well, insufficient numbers of healthcare workers appear comfortable in managing adolescents and/or their mental health problems [23], and appropriate medical services may not be readily available to adolescents when and where they want to be seen [23].

### Artificial intelligence and adolescents’ mental healthcare in primary care

To improve PCP delivery of mental healthcare, integration and cooperation with mental health experts has been sought [24, 25]; along with increased continuing medical education [26] and monetary incentives [27]. Novel technologies such as artificial intelligence (AI) have created potential opportunities for assisting PCPs with mental healthcare [28]. AI represents that discipline of engineering and computer science dedicated to developing intelligent machines [29], and is seen as a method for facilitating, augmenting, and/or enhancing human work [30, 31]. It has the potential to improve healthcare services [31], including automating medical devices [30], administrative planning [32], and resource management [33] to support prevention, screening, diagnostics, and treatment [30].

Healthcare has been slow to adopt/implement AI compared to other service sectors, especially in the area of mental health [34] despite its promise in supporting PCPs involved in adolescents’ mental healthcare [35]. Based on a scoping review we conducted on the use of AI in adolescents’ mental health care [36], we are unaware of prior research exploring PCPs’ needs for and challenges with AI systems that support adolescents’ mental health. The goal of this research was therefore to identify a): PCPs perceived challenges in providing adolescents’ mental health care and b) PCPs’ perspectives on the AI’s potential for assisting them in this care.

## Methodology

### Design

We adopted a qualitative descriptive design as it was the most suitable method for obtaining straightforward, minimally theorized responses from PCPs on a topic that has received little attention like application of AI in adultescents mental health care [37].

### Eligibility criteria and participant recruitment and consent

Using purposeful sampling [38], Montreal-based English- speaking family physicians and primary care pediatricians known to routinely provide adolescent healthcare were sought for this study. A list of 35 potential participants was generated by consulting with physician leaders in pediatrics and family medicine. Email invitations were sent to them, asking for their participation in focus groups (FGs) exploring perceived needs of PCPs for AI systems focused adolescent mental healthcare. A follow-up telephone call was made 10–14 days later using office numbers located on the public website of the Collège des Médecins du Québec. Those not reachable received a second email. Interested FG participants were sent electronic consent forms that described an on-line FG lasting between 60 and 90 min, audio-visually recorded using Zoom software, with 256-bit End-to-end encryption making it impossible for anyone but the interviewer and interviewee to access or understand interview contents [39]. There was no compensation for study participation, and an online polling app was used to establish an acceptable date and time for the focus groups. Prior to the FG, participants were informed of their right to leave the session at any time, and that their responses would be confidential, anonymous, and used for descriptive purposes only. All materials generated by the study were stored on a password-protected McGill University OneDrive server. The study received ethics approval from the McGill University’s Faculty of Medicine and Health Science’s Institutional Review Board (IRB) prior to commencement (A01-B12-21B).

### Focus group activity

Due to COVID-19 pandemic mitigation measures, we were unable to hold face-to-face focus group sessions and had to conduct them online instead. Participants received written log-in instructions and completed an on-line demographics questionnaire prior to the session [40]. To ensure that participants began discussion with some common general knowledge about AI, the FG started with a brief presentation given by facilitator #1 (PG) (See Appendix A). The presentation consisted of a few examples of AI used in non-healthcare situations, having been previously piloted in talks on AI given by PG, independent of this study. Feedback was positive on their educational value. FG discussion then followed a semi-structured interview guide using open-ended questions created by the research team (Appendix B). Discussion was stimulated by probes and requests for elaboration [41]. Zoom software recorded and transcribed the discussions into documents for later thematic analysis. Facilitator #2 (MJY) participated in the FG primarily as an observer and for support of the first facilitator. Immediately following the FG the facilitators engaged in a half hour debriefing on the FG process and the data it generated [42].

### Data analysis

Live transcriptions were reviewed and edited. Punctuation marks and symbols were inserted to indicate speech pauses, participant voice tone and level of engagement. This facilitated transfer of the participants’ feelings and intent through text (Table 1). All members of our research team (SAR, MJY, AMA, PG) analyzed the data during twelve two-hour sessions following the six phases of thematic analysis recommended by Braun and Clarke [43, 44]. Phase one focused on data familiarization, internalization and immersion through repetitive transcript reading, thus enabling reflection on participant comments, and identification of recurring concepts and areas where participants agreed or disagreed. In the second phase, we initiated the coding process whereby chunks of data were labelled systematically to facilitate the identification of patterns and themes. A *priori* codes that focused on the main questions in the FG guideline were precisely defined to ensure their systematic application to the data (Appendix C). Additional inductive codes were also identified and defined based on the “close examination of the data without attempting to fit the information to pre-existing conceptions or ideas from theory [41].”

**Table 1:** Symbols used in transcription verbatim

Punctuation mark / symbol	Indication
(? time)	The exact timing of a phrase or sentence couldn’t comprehend owing to low audio quality
…	A long pause or a sudden shift in sentence
Bolding and underlining words/phrases	Words/phrases expressed loaded with emphasis and significantly louder volume
[]	Added term to the statement to better express the idea of the participant
“ ”	A direct verbatim quote from the participant

The third phase was theme development. Themes, defined as “recurrent notions that may be utilized to summarize and organize the variety of subjects, opinions, experiences, or beliefs expressed by participants” [41], emerged through the iterative review of codes and participant perceptions of PCP needs and challenges in using AI systems to support adolescents’ mental healthcare.

In the fourth phase identified themes were iteratively evaluated for meaningful coherence. Possible links or correlations between themes were sought. A thematic map evolved for describing PCPs’ perceived needs and challenges in adolescents’ mental healthcare using AI systems. The fifth and final phase involved the definition and justification of themes and the generation of sub-themes. To ensure rigor, the five criteria for trustworthiness in research proposed by Lincoln and Guba i.e., credibility, transferability, dependability, confirmability and authenticity, were followed [45]. An ‘Audit Trail’ was created detailing the process of data collection, analysis, and interpretation. Participant wording, including raw quotes, was retained to provide authenticity. The research team’s thoughts about coding were documented, rationale for merging codes was developed, and extensive discussions took place regarding the meanings of various themes and codes. Supplementary information can be found in the Appendices.

We resolved disagreements throughout these phases by using clear criteria and procedures for coding and categorizing the data. We held regular team meetings to discuss interpretations and address any discrepancies. We employed peer debriefing and consistency checks to validate our findings and ensure consistency across the dataset. When disagreements arose, we documented them, maintaining reflexivity and transparency about our biases and perspectives. We utilized software tools (e.g., Microsoft Word) to facilitate collaboration and analysis. Through this iterative process, we revisited the data multiple times, seeking expert consultation when needed. Our efforts resulted in a rigorous analysis, ultimately leading to credible research findings.

## Results

**Fig. 1:**
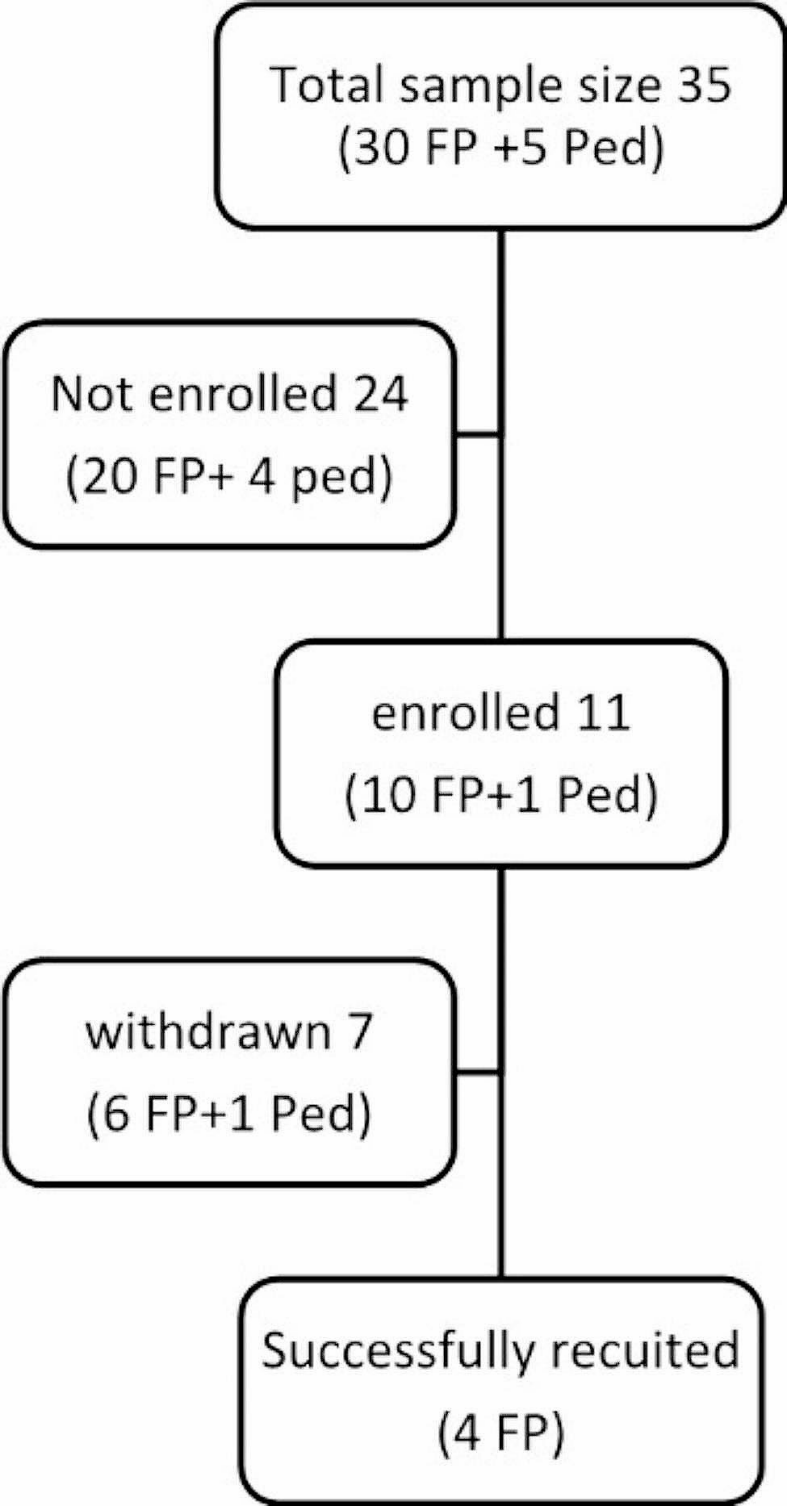
Participant recruitment process and results. (FP: Family Physician; Ped: Pediatrician)

Figure 1 summarizes the outcome of participant recruitment. While the study protocol aimed to recruit 12–18 participants into 2 to 3 FGs, only 11 of the 35 physicians approached enrolled in the study. Prior to the FGs taking place, seven participants withdrew from the study due to unexpected work commitments related to the COVID-19 pandemic. We consequently implemented a single FG comprised of 3 female and 1 male participants. Their characteristics are summarized in Table 2, with years in medical practice ranging from 6 to 40, and the proportion of their estimated clinical time caring for adolescents in an ambulatory setting varying from 5 to 20%. Table 3 summarizes the outcome of our analysis which identified 5 major themes: (1) Challenges of giving adolescent care in ambulatory settings; (2) Perceived features and characteristics of AI systems; (3) Potential applications of AI systems; (4) Possible negative aspects of using AI systems; and (5) PCP’s perceived requirements for use of AI systems.

**Table 2:** Demographic characteristics of the participants

Participants	Gender (Male/Female)	Clinical experience in medical practice (Years)	Participants’ ambulatory care time spent on adolescents (%)
P1	Female	6	10–15
P2	Male	13	12
P3	Female	40	5
P4	Female	22	20

**Table 3:** Themes, subthemes and sub-sub themes in adolescents’ mental healthcare in the primary care setting

Themes	Subthemes	Sub-Sub themes
1. Challenges of giving adolescent care in ambulatory settings	1.1 Fostering and maintaining a relationship	1.1.1 Difficult due to level stage, characteristics, peers, parents, and technology
	1.2 Adolescent care is time-consuming	1.2.1 Physicians’ time management
2. Perceived features and characteristics of AI systems	2.1 Perceived benefits to PCPs and healthcare system by easing activities, and providing efficiencies and facilitation	2.1.1 Easing access to the resources 2.1.2 Easing data collection and storage (e.g., patients’ demographic information or lab results) 2.1.3 Handling large volume of data 2.1.4 Cost efficiency 2.1.5 Time efficiency 2.1.6 Taking patients’ history 2.1.7 Treatment planning 2.1.8 Facilitating relationship with patients 2.1.9 Efficient questioning 2.1.10 Interactive patients’ questionnaires 2.1.11 Promoting medication adherence
	2.2 Credibility	2.2.1 Negative Credibility 2.2.2 Positive Credibility
	2.3 Potentiality for benefit varies by user	
3. Potential applications of AI systems	3.1 Clinical care	3.1.1 Administrative support 3.1.2 Patients’ triage 3.1.3 Decision support 3.1.4 Establishing/helping to validate diagnosis 3.1.5 Tracking patients’ progress and improve patients’ adherence to treatment 3.1.6 Identifying community resources to which patients may be referred i.e., psychological management through CBT
	3.2 Obtaining and analyzing data	3.2.1 Algorithm-generated questionnaires 3.2.2 Questioning based on high yield queries suggested by AI 3.2.3 Identifying patients’ possible associated conditions 3.2.4 Distilling patients’ information for use in a particular clinical context 3.2.5 Finding and Interpreting variables that might suggest red flags
	3.3 Medical education and research	
4. Possible negative aspects of using AI systems	4.1 Profession threatening	
	4.2 Trust issues (Mistrust or Distrust)	4.2.1 Performance Accountability
	4.3 Misinformation or disinformation	
	4.4 Lack of human connection	
	4.5 Diminished clinical competency	4.5.1 Negative impacts on knowledge, attitudes, and skills
5. PCP’s perceived requirements for use of AI systems	5.1 Need for education on AI	5.1.1 Population in general 5.1.2 Continued professional development 5.1.3 Medical residents and students
	5.2 Need for user-friendly AI systems co-developed with clinicians, supported by “just in time” technical support	5.2.1 Easily operational and seamless 5.2.2 Relevance and meaningfulness to practice 5.2.3 External incentives (credited CMEs, financial) or personal reward and interest in research and topic
	5.3 Need for AI regulatory bodies	5.3.1 Issues of confidentiality, privacy, trust, and liability 5.3.2 Need for having a framework/guideline to increase safety of AI systems 5.3.3 Need for research into AI systems’ validation and its applications
	5.4 Financial implications of AI use	

### Theme 1: challenges of giving adolescent care in ambulatory settings

This theme represents the difficulties that PCPs face in providing outpatient care to adolescents and was separated into two subthemes: (1) Fostering and maintaining a relationship; and (2) The time-consuming nature of adolescent care.

#### Fostering and maintaining a relationship

PCPs noted challenges related to establishing relationship with adolescent patients. Among these were problems building the necessary trust to initiate a relationship due to adolescence stage of development, personal characteristics, peers, parents, as well as technological barriers. An example of the latter relates adolescent to particularities in cell phone use that impedes good communication:

*A lot of times because of confidentiality, you’ll have their* [adolescents] *cell phone numbers on file, but they’re in school and they won’t pick up the phone and they don’t call back.* (P1)

Despite the difficulties of establishing a longitudinal connection with adolescents, a number of PCPs emphasized their desire for mutual understanding, continuity of care, and better health outcomes over time.

*If you follow your patient longitudinally* [starting] *as a child* [you build a] *foundation, so that when they have a problem, you have a relationship already.* (P2)

Various pragmatic strategies were proposed to maintain the doctor-patient relationship. For example, “normalizing certain behaviors” was important in every meeting with adolescents. This includes clearly defining issues such as “confidentiality” so that adolescents feel comfortable in expressing themselves freely, resulting in a more stable and trusting relationship. At the same time they recognized that many adolescents prefer computers and tablets over human doctors, giving them more space to open up about their mental health needs:

*Having data on your patient beforehand is important and there’s a lot of data that we could ask for* [however, adolescents aren’t] *forthright with their answers when you do it in person; but a* [online] *questionnaire that’s done objectively can allow them to feel like they’re not being judged when answering those questions.* (P2)

Some PCPs identified the positive and negative impacts of parental involvement in dealing with adolescents’ noncompliance in providing accurate information to the doctor. Various challenges included legal issues, and struggles around parental control, including their reluctance/inability to give their child autonomy. Some parents are “overprotective” and “too present,” making adolescents uncomfortable/hesitant in expressing themselves at appointments: [If] *the parent sees the questionnaire* [history intake questionnaire], *either before or after it’s filled out, they may discourage the teenager from filling it out truthfully.* (P2)

PCPs also highlighted adolescent’s’ sex and gender, family background, culture, peers, and habits as important influences in doctor-patient encounters that need to be managed. Some of these require PCPs to use a new lexicon.

*Something I find very challenging in dealing with adolescents is the complexity of the social environment: [for example] different family backgrounds…and we don’t talk on the same level as we talk with adults. We have to use another vocabulary.* (P3)

#### Adolescent care is time-consuming

PCPs indicated that more time is required to care for adolescents due to the complexity of their life cycle issues, noting the utility of self-administered questionnaires and multiple visits before the bigger picture becomes clear. One sub-theme that arose related to time management in the context of adolescent mental health care.

*“I will have to see this person, maybe a second or third time, before I start to get the picture…That’s why we use questionnaires is for adolescent patients that they fill out before they come in … But we have to invest a lot more time into these patients* [Adolescents]” (P3).

A solo private practitioner expressed frustration about time spent linking adolescents to supportive community services, adding that those working in public practice centers may have fewer problems accessing multidisciplinary programs. PCPs also noted their job is to “identify the problem” and “provide care” rather than coordinate and organize access to supporting resources, as they are commonly not compensated for these time-consuming tasks.

*My job is to figure out what the problem is and what I should do, but then to go and find out where the fax number is and who might know what resources might be available…that’s challenging, time consuming and below our pay grade.* (P2)

### Theme 2: perceived features and characteristics of AI systems

A variety of benefits were suggested by PCPs related to creating efficiencies in accessing mental health resources, and collecting, storing, and handling large volumes of data (e.g., patients’ demographic information or lab results); time and costs efficiencies related to patient history and questioning, and treatment planning, and support with facilitating relationships with patients, promoting medical adherence and organizing interactive patient questionnaires.

Participants felt that AI systems might increase access to primary care resources and help optimize resource utilization. One participant noted: [Having robots to do Cognitive Behavioral Therapy] [maybe] *more cost effective, and* [easier] *to access than then having a real person as the bot has zero bias … and it’s free!* (P2)

Cost and time efficiencies and more efficient questioning were also mentioned as potential benefits of AI systems for patients and PCPs in the context of referrals to specialists, which prolong patient treatment and are a “cost-drain” on the system.

AI’s capacity to handle enormous amounts of data was another benefit, with one participant reflecting that easier access to information may allow PCPs to better address adolescents’ mental health and other primary care concerns.

*Family Medicine is a very challenging* [field]*… we need to know a little bit about everything*…[by contrast] *AI it’s just hungry for data, it will just never tire!* (P2)

One respondent expressed that AI might facilitate collaboration between the PCP and multidisciplinary healthcare team (including specialists and social workers), saving time and making adolescent visits more efficient. Another PCP suggested using AI to take histories that involve sensitive questions to help doctors who feel uncomfortable asking certain questions.

Several PCPs further noted that AI systems have the potential to tailor the application of available therapeutic resources to the adolescents’ specific objectives and requirements, resulting in a personalized treatment strategy for each patient: *AI could be useful to more specific treatment plan*, [and] *give us real concrete targets.* (P2)

Another type of benefit is AI’s potential in enhancing the patient-provider relationship. PCPs felt that since adolescents are comfortable engaging with technology, supporting the doctor-patient relationship with AI systems (e.g., self-administered questionnaires on a laptop rather than face-to-face inquiry) would make it less threatening.

*Adolescents are more comfortable answering to a computer than a doctor. (P4) … Can the machine* [AI system] *persuade* [instead of compelling patients by a human doctor], *a patient to take a treatment?!maybe! (P3)*

However, uncertainty was also expressed. Several participants struggled with whether AI systems would be accepted by patients and considered a credible source of support. An example of negative or low credibility was voiced:

*We can still talk and try to persuade the patient … I think that if the patient has confidence in us, they may agree to the treatment. Now, would they have the same reaction to a machine* [AI system]*?!* (P3)

Offsetting this viewpoint was the potential for AI systems to learn and direct patients more positively than doctors: *AI can learn one day to do that* [treatment adherence], *and to do it better than the best doctor*. (P2)

### Theme 3: potential applications of AI systems

Several applications of AI systems were identified by PCPs in the areas of clinical care, obtaining and analyzing data, and medical education and research. In terms of clinical care, several PCPs discussed how AI systems might be useful in facilitating administrative tasks (e.g., appointments, paperwork for in-office and out-of-office care, patient discharge) and case management which may involve collaboration with interdisciplinary services.

*I’m at a severe disadvantage working alone, compared to those who work at the clinic Y* [public clinic]. [it’s] *now maybe hard to access certain services. I’m sure you all have challenges in terms of bookings, whether it’s* [for] *social workers, nutritionist, psychologist, and AI can help here.* (P2)

PCPs also noted AI systems might aid decision-making by providing PCPs, adolescents, or other individuals with knowledge and person-specific information filtered or presented at appropriate times to enhance mental health diagnosis and treatment planning.

*Machine learning can be used as a decision support system* [by utilizing] *computable data* [to] *make* [diagnosis and treatment] *recommendations*…*specific* [for] *adolescents*. (P2)

Given the complexity of accurately diagnosing adolescents’ mental health problems, PCPs viewed AI systems favourably to assist with diagnosis and its validation.

*It* [AI], *really helps in terms of diagnosis. We don’t want to miss something that serious when you have to make critical decisions.* [For example], *should I send this patient home or to ER* [Emergency Room] *because the stakes are quite high in terms of self-harm?!* (P3)

Participants noted the potential utility of AI in assisting with the complex and time-consuming process of patient referral, including consultation with a medical colleague, home care, organizing a visiting nurse, or finding community resources including CBT services. Indeed, one participant suggested how CBT provided by an AI robot outfitted with forms, characters, and faces to replicate human interaction with patients would be more cost-friendly, bias-free, and engaging for adolescents.

*AI could be useful, give us real concrete targets in terms of what’s out there in our community… it could know, for example, that there’s five places left on this support group starting next week. There are chatbots for this purpose* [CBT], *and work* [based] *on 3D avatars, facial expressions and interacting with a virtual person.* (P2)

A second application of AI relates to data collection and analysis to facilitate adolescent mental health assessment and support. PCPs believed that AI-assisted medical robots could generate and analyze patient information to generate answers to clinical questions efficiently and cost-effectively and help them have a more focused practice. As one participant remarked:

*…I dream of having that as an AI bot or system to tailor the questions we need in more detail and skip over things you don’t need a detail to really concentrate more on certain aspects of the patient* (P2 and P3).

A PCP (P2) with higher AI knowledge noted that Natural Language Processing might automatically summarise patients’ information during/after a visit, while AI models could be used to evaluate patients’ cardiovascular risk factors instead of a doctor doing it manually in the office. PCPs also recognized AI’s potential to identify red flags and abnormalities in patients’ data:

Machine learning can be used for a decision support system, where it may pick up on some of the computable data that comes out of an interaction or questionnaire… it may raise some flags, and say: “*have you considered this diagnosis or that?”* (P2).

A final area of application was in the area of medical education. PCPs noted the potential use of AI systems in continuing medical education (CME) to suggest courses and training based on practitioners’ interests and practice composition. Also, they saw potential in such systems to handle and compare trials of varied sizes, diverse sample populations, overlapping research topics, and store previously collected data.

[AI may] have *a role in recommending* [CME] *courses that you might find interesting. A recommendation like a Netflix for CME, … based on your history of CME or the configuration of practice.* (P2) - [Using AI] *for research, if we can have this* [collected data] *piled somewhere, and somebody would like to have research done*, [it] *would be so easier.* (P4)

### Theme 4: possible negative aspects of using AI systems

Despite perceived benefits and applications of AI, several negative aspects were noted.

One area of concern was the implications of AI for professional practice whereby AI might compete with or replace highly skilled clinicians given potential capacity to perform more sophisticated tasks.

The issue of trust in AI was also raised. PCPs referred to the risk of lack of confidence in the truth, validity, accountability, or effectiveness of using such systems; impressions based either on an individual’s intuitive/gut response (mistrust) or real experience (distrust) are illustrated in doctors’ comments as follows.

*For ‘diagnostics’ I think the fear was always it* [using AI] *was wrong! I don’t want to act on something that just calculated things wrong! … it* [AI system] *can tunnel vision you even though you try to use your clinical judgment.* (P1)

Given the widespread presence of misinformation (incorrect information) and disinformation (intentional spreading of misinformation) found in social media, one participant worried about what controls there would be on AI systems used in healthcare:

*What I’m hearing is AI is gonna replace everything? How can it replace us eventually one day?!*

A further worry was that AI systems, as artificial beings, lack passion, enthusiasm, worry, empathy, and face-to-face emotion, all of which are critical dimensions of good clinical practice:

*The human aspect of medicine is very important. We don’t want the patient to feel they treated by a robot. That is the disadvantage* [of AI systems]! * It is very important that patients feel empathy…*[Hence] *patients feel a lot better*. (P3)

Concerns about the potential for diminished clinical competency were also expressed. PCPs worried that using AI systems might undermine their medical and patient communication skills for *if you don’t use* [your skills], *you’re not going to know how*. (P1)

### Theme 5: PCP’s perceived requirements for use of AI systems

PCPs were asked to discuss how they envisioned their needs using AI systems in adolescents’ mental healthcare. Education on AI was identified as a priority for their own continued professional development as well as for those in training e.g., medical residents and students. While PCPs felt the need for CME training on AI to learn about new and developing areas in their fields, concern was expressed about how demanding, time-consuming, or relevant such courses might be.

For residents and medical students, training on AI was deemed vital, part of the mandatory curriculum, and perhaps as a new discipline:

*I think this [training in AI] should start in medical school! There should be even a specialty such as “medical informatics.” (P2)*.

At the same time, PCPs stressed the importance of governance and regulation to ensure AI systems were used ethically and effectively.

*Definitely I think it makes a lot of sense to have some kind of regulation, when that we’re using* [AI systems] *therapeutically!*

Particularly vital was preventing potential ethical or physical harms arising from the use of AI systems in the adolescent age group. Because today’s adolescents are more tech-savvy, they might express themselves better via computers, resulting in a more confidential visit. PCPs were therefore concerned about AI-driven privacy breaches and acknowledged the importance of protecting patients’ data and anonymity. However, they struggled with the nuances of perceived responsibility (doctors vs. AI) when utilizing such systems.

*There are all kinds of privacy issues. All it takes is one breach and people will lose confidence* [in AI] *… if a doctor making a decision that was supported by AI; who’s responsible?! It’s clear that we are! So, can you use that as a defense in front of a judge?! I don’t think that’ll hold up* [in court], *but it’ll probably be used as a defense at some point!* (P2)

For this reason, several PCPs emphasized that they should be held accountable for possible erroneous AI-assisted care, and suggested that frameworks or guidelines and human supervision be put in place to ensure that AI systems “do not harm”:

*Once technology* [AI] *is mature…*we [need] *the right framework in place to make it safe for patients. I certainly don’t want to be responsible for people committing suicide because of my chatbot! We’re going to be supervising these systems and making sure that are working.* (P2)

PCPs also noted the importance of ensuring that AI systems employ a friendly user interface, so non-technical users with limited AI understanding might rapidly attain mastery, sync it with their practices, and get on-demand, “just in time” technical support.

*Technical support is very important… a good instruction manual that I can understand … and user friendly so if I run into difficulties … I can call someone* [for] *help*. (P3)

A number of participants expressed an interest in contributing to AI systems’ design and development if deemed helpful to patients. Three important features were deemed essential in AI system roll-out; that AI systems be easily operational and seamless; relevant and meaningful to practice; and that uptake be incentivized through credited CMEs, as well financial and professional opportunities. On the issue of incentivization, PCPs felt it necessary to offer external incentives and opportunities including to engage in AI system design and development.

*I don’t think that you’ll get a lot of capture with volunteering*. *It has to be incentivized, either through credits* [CME credits], *interest research or* [some form of payment]. (P2)

Finally, the financial implications of adopting AI systems were stressed. Participants noted that that PCPs were unlikely to use AI systems if initial and operating expenses were too costly.

## Discussion

This research yielded insight into how PCPs see AI systems influencing mental healthcare for adolescents. Of note were the many complexities limiting the adoption of such AI systems by PCPs yet the opportunities that it presents if necessary support was provided. Each theme is discussed in turn.

### Ambulatory adolescent care challenges

Adolescents present challenges in the delivery of primary care [46] and our PCPs identified obstacles that include biological, psychosocial, cultural, peer, and familial factors. These are consistent with the published literature [47, 48], and help contextualize adolescents’ fear of stigmatization [49] and concerns about privacy [18, 50] that reduce the likelihood of seeking care and adhering to recommendations [47–52]. It has been suggested that creating and sustaining solid connections between doctors and adolescent patients is crucial for reducing barriers and for developing favourable lifelong attitudes to healthcare [53, 54]. The present study highlights PCPs awareness of the need to build rapport, trust, and effective therapeutic relationships between themselves and adolescents.

### Features and potential applications of AI systems

The PCPs in this study believed AI has the potential to ease clinical burden by increasing efficiencies, and facilitating patient interface with the healthcare system. This aligns with previous research indicating that AI may be useful in enhancing PCP productivity, accuracy and efficacy [29, 55], and generating more reliable data collection and more accurate diagnoses, especially when evaluations are expensive or time-consuming [56]. The views of our study’s PCPs align with experience in laboratory medicine where AI has improved data access and real-time interpretations of test results, leading to improved patient care [57, 58]. The analysis and interpretation of digital information in mental health offers potential for preventing mental health problems, identifying new concerns, suggesting tailored and targeted therapy, monitoring relapse, altering prognosis, and identifying relevant community resources [59, 60].

Participants noted AI can facilitate clinical decision-making, in line with research illustrating feasibility of AI-enabled decision support systems in clinical contexts, such as in choosing antidepressant medications [61–63] or in medical triage [64]. These applications can facilitate patient flow and streamline needs for clinical staff [65]. Examples of AI to support adolescent mental health include Kids’ Help, an online AI platform which triages users who contact the crisis text line [66], or by acting as a patient intake coordinator performing screening tests before linking them to a physician [67].

AI-assisted online behavioural therapy and conversational chatbots may be a cost-effective and engaging treatment planning alternative [68]. CBT online chatbots, such as Sara [69] and Woebot [70], replicate common communication methods, and in college adolescents, reduce depression and anxiety, and boost adherence to treatment and psychological management.

Our results support the view that AI offers potential for providing administrative support. Recent research has revealed that AI can automate repetitive, time-consuming tasks like paperwork and administrative information processing [71, 72]. AI can also assist clinicians in monitoring their patients’ health between visits, thus freeing them to provide more focused care [73, 74].

Most AI applications for mental health are still in the research and development stage and have not been scaled up for clinical practice or patient use [75]. If responsibly developed, the implications of integrated AI in mental healthcare are exciting, with the potential to support both operational and clinical functions for the benefit of both physician and patient [76].

### Risks associated with AI systems

We found concerns about the “credibility” of AI amongst some participants who questioned whether AI was capable and credible enough to suggest a treatment plan to patients. Within published literature there are conflicting studies [77]. For example, wrong labelling of data samples used to fit an AI method [78] may generate erroneous or biased interpretations and subsequent recommendations that may cause harm [79, 80]. Other research has demonstrated that systems may have an unlimited capacity to learn, with consequent potential to help patients [81]. Participants also expressed concern that the introduction of AI might diminish their professional skills and competencies, or replace them as providers—perhaps reflecting their own lack of knowledge about AI [82–85]. This suggests that before integrating AI into clinical practice, it is necessary to determine what tasks can be shifted without jeopardizing the existing quality of care and PCPs’ ability to continue to practice.

Even though AI systems in healthcare aim to replicate or improve physicians’ efficiency [86], replacing doctors’ tasks with technology risks reducing emotional touch as mirrored in our findings [58, 87, 88]. Study participants stressed the importance of face-to-face human interaction, noting that AI can’t replace humans in delivering empathetic care. Therefore, AI architecture must support a care model that is compassionate and competent in responding to patient needs [86].

### Requirements and conditions for using AI systems

Integrating AI into adolescent mental health care might raise ethical considerations [89] such as privacy, consent, trust, liability, and issues associated with algorithmic decision-making [90] which were among issues raised by our participants. Some concerns also revolves around the confidentiality of sensitive personal health information, and the safeguarding of it against unauthorized access or misuse [91]. Informed consent may be particularly challenging for adolescents as they may need assistance or support to fully understand the implications of sharing their data or engaging with AI-driven interventions [92].

As one considers ethical issues the need for regulatory bodies may increase in direct proportion to the capabilities and accessibility of AI in mental healthcare in order to minimize breaches caused by either the AI or physicians [93]. Supporting this need, study participants emphasized the importance of governing authorities to ensure that AI systems are safe. Medical, ethical, and legal standards help regulate the doctor-patient-family relationship, prioritize transparency, and engage in ongoing dialogues with adolescents, caregivers, and healthcare professionals to navigate these ethical challenges responsibly [94]. Our participants emphasized that trust and privacy are critical issues surrounding AI that might impede or facilitate the doctor-patient relationship. For instance, the Canadian Protocol checklist i.e., an ethical framework for AI applications in mental health [95] and the Canadian AI Algorithmic Impact Assessment (an open-source platform) [96], help AI decision-making system developers mitigate privacy, transparency, health, and bias risks. This approach will allow us to harness AI’s potential while upholding the rights, dignity, and well-being of adolescents in mental health contexts. Striking a balance between the benefits of AI in improving mental health outcomes and protecting young individuals from exploitation or undue influence is crucial [92].

It is noteworthy that FG participants did not raise any concerns about inherent biases within AI algorithms that could perpetuate disparities in mental health care, especially for underrepresented, racialized, or marginalized groups., issues that have been described in the literature [97, 98]. These biases can reinforce harmful stereotypes, leading to problems in access to care, misdiagnoses and inadequate treatment [98, 99].

A growing literature urges that AI developers disclose what type of data is gathered, who has access to it, how the information will be used, and what measures are in place to prevent bias and harmful use of the data [100]. Our participants noted that PCPs could be held accountable for outcomes arising from employing AI in their practices. Biased algorithms perpetuate disparities and hinder the development of tailored interventions, ultimately impacting the well-being of adolescents [99].

Study participants highlighted the importance of education about AI in the healthcare sector. In business and science, the use of AI is relatively well known and accepted as a means of enhancing user experience, work efficiency, and job opportunities [101]. However important investments in “digital literacy” may be required to scale-up AI deployment in healthcare [102]. AI education for physicians, residents, and students, including its potential incorporation into medical school and residency curricula [103] were suggested by our study participants, although few such initiatives have been described [104, 105]. These doctors also stressed that training could ensure safe application of AI in patient care [106]. Finally, participants noted their preference for user-friendly AI systems that are co-developed with clinicians and serviced with timely support. These requirements are in alignment with the literature exploring best practises in introducing innovative technologies into healthcare [107].

### Strengths and limitations

This study used exploratory qualitative inquiry to examine primary care providers’ perceived challenges and needs for AI systems to support adolescents’ mental health. A limitation of the study was the small number of participants, who were unable to adjust their schedules for participation due to the COVID-19 pandemic and increased workload. However, the participating group was highly interested and vocal about the study topic, and we included providers with varying levels of experience in adolescent care. Since qualitative research is dependent on the quality and depth of information and not necessarily on the number of participants, the PCPs in this study provided rich data for consideration.

The COVID-19 pandemic eliminated face to face encounters in the FG and conducting a qualitative study using online meeting software presented some challenge. To maintain data confidentiality, we audio-visually recorded the FG on personal computers rather than utilizing cloud-based online storage. While occasional inconsistent internet connections or voice cuts were experienced, the overall virtual environment did not appear to compromise data collection. Participants seemed comfortable discussing their views and experiences from their preferred location without the need for travel.

## Conclusion

This research provides insight into PCPs perceptions of AI systems and their application for adolescent mental healthcare. While a range of convergent and divergent attitudes were expressed, most participants were enthusiastic about the potential for AI systems in improving quality and scope of primary care. While this study provides groundwork for assessing the utility, applicability, and possible effectiveness of AI in adolescents’ mental health care, larger surveys are suggested for greater clarity on these systems. We also suggest exploration into adolescents’ perspectives on integrating AI into their own mental healthcare. The COVID-19 pandemic has demonstrated primary care can benefit from technological solutions to ease overstretched healthcare resources. Successful application of AI will depend on proper AI training for both current and future PCPs. Additionally, robust regulatory frameworks are essential to ensure that ethical standards are upheld through the development and use of AI systems. These measures will help guarantee the safe, effective, and responsible integration of AI into healthcare practices.

### Electronic supplementary material

Below is the link to the electronic supplementary material.

Supplementary Material 1

## Data Availability

Datasets used and/or analyzed during this study are available from the corresponding author upon reasonable request.

## Abbreviations

AI: Artificial Intelligence
CBT: Cognitive Behavioral Therapy
CME: Continuing Medical Education
FGD: Focus Group Discussion
FG: Focus Group
ML: Machine Learning
NLP: Natural Language Processing
PC: Primary Care
PCP: Primary Care Physician
PCPs: Primary Care Physicians
