# Supplementary material for: Primary care physicians’ perceptions of artificial intelligence systems in the care of adolescents’ mental health
Source: BMC Prim Care. 2024 Jun 13;25:215. doi: 10.1186/s12875-024-02417-1 (PMC11170885; doi:10.1186/s12875-024-02417-1)
Supplement: Supplementary file 1 — Supplementary Material 1 [file 12875_2024_2417_MOESM1_ESM.docx]

**Appendix A:** Artificial Intelligence Non-Medical Uses and Examples- Presented as PowerPoint slide slides to the participants

(Narratives related to every slide is written following each slide)


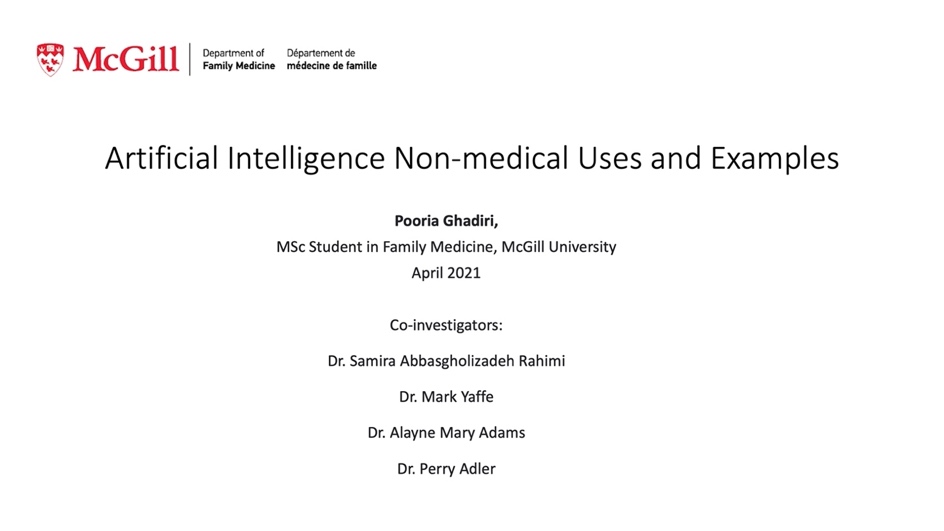


Now, on behalf of all team, today I am going to present to you some examples about “Artificial Intelligence Non-medical Uses” in 7-8 minutes in order that everyone in the focus group has the same understanding of what AI is, so that we can then discuss your ideas about AI in the care of adolescents' mental health.


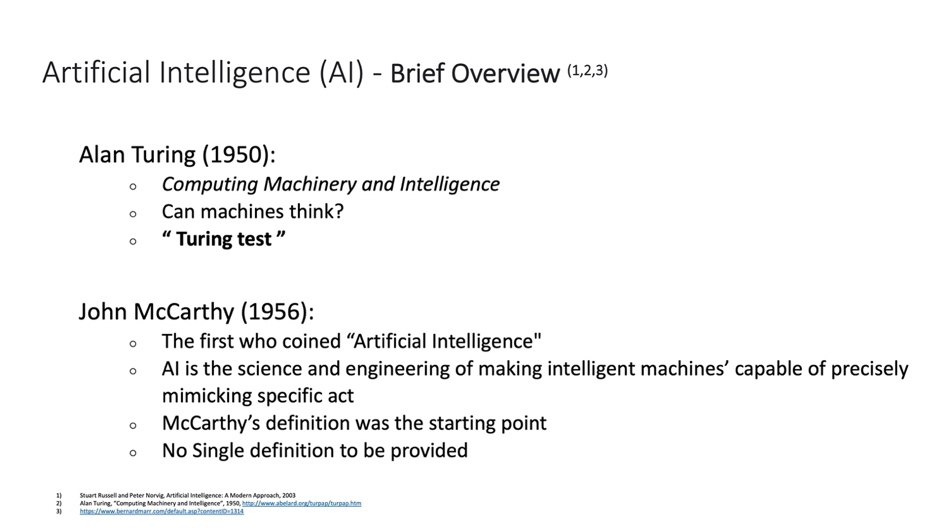


In 1950 English Mathematician Alan Turing, one of the very first AI pioneers, published a paper entitled “Computing Machinery and Intelligence” which opened the doors to the field that would be called AI. The paper itself began by posing the simple question, “Can machines think?” Turing proposed a method for evaluating whether machines can think, which came to be known as the ‘Turing test’. The ‘Turing test’ takes a simple pragmatic approach, assuming that a computer that is indistinguishable from an intelligent human actually has shown that machines can think. Years later, the term Artificial Intelligence was first coined by Dr. John McCarthy at Dartmouth conference, Hanover, New Hampshire, USA in 1956. He defined AI as ‘the science and engineering of making intelligent machines’ capable of precisely mimicking specific act! It is necessary to mention that the given definition was just a starting point and there is not really a single definition to be provided.


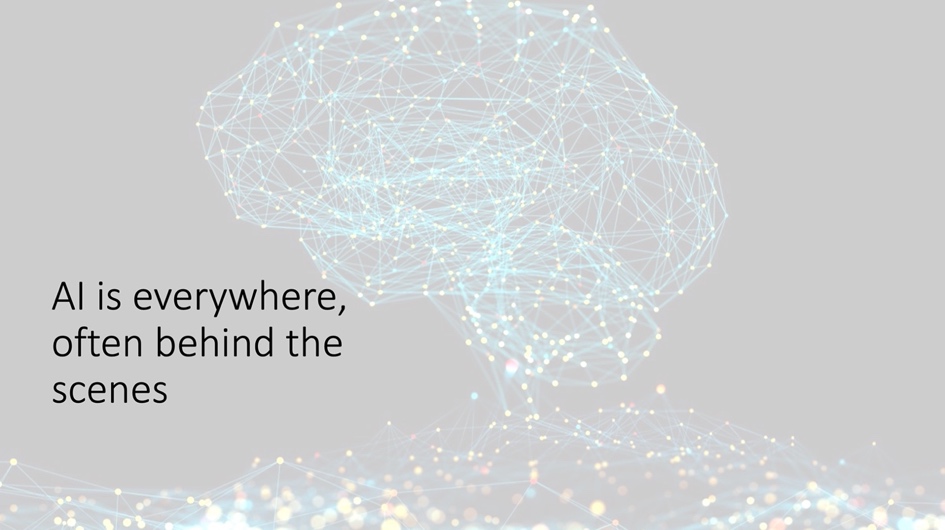


Sometimes, when we hear the word AI, we might think that this has nothing to do with us or something we don’t encounter within our day to day lives! However, the truth is quite the opposite. Every single one of us meets AI multiple times each day. Even if we aren’t aware of it, AI is at work, often behind the scenes, as we go about our everyday lives. For instance:


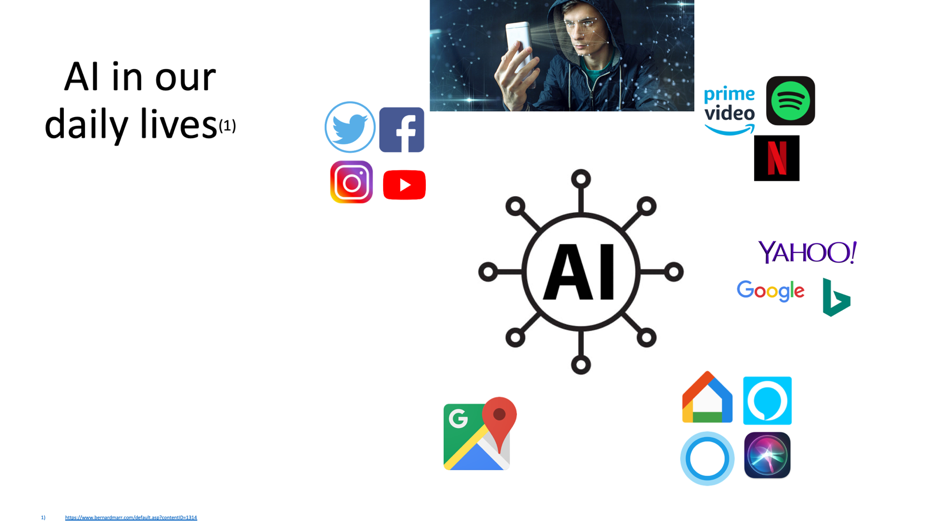


**Face Recognition:** When people wake up in the morning, picking up their iPhone, and it opens up automatically by recognizing their face, this is basically AI in practice which scans the face and grants a subsequent access to the phone content. **Recommender Systems:** When users check on social media feeds such as Facebook, Twitter, Instagram, AI is used to supply users with information close to their history and interests. Such companies also use AI to further protect their users by screening fake news, fighting to cyberbully running in the background without people necessarily knowing. Similarly, companies such as Netflix, Spotify, and Amazon use AI to recommend content related to their users' tastes by learning their interests and behaviors. Google search engine serves users based on what it knows about them and what are the themes in which the users are interested. This is all driven by AI. Every search result is now personalized to the users, thanks to the AI working in the Google background. **Speech Recognition:** Apple Siri, Amazon Alexa, Google Home, Microsoft Cortana use AI to understand what the users are actually saying to them and generating reasonable answers. **Natural language processing and natural language understanding:** Chatbots use Natural Language Processing to understand and communicate with humans. Known as a NLP, this AI technology focusses on understanding on how humans communicate with each other and how we can get a computer to understand and replicate that behaviour. In business setting, for instance, chatbots are quickly finding their place in customer services. It is expected that in a few years, chatbots will power 85% of all customer service interactions.


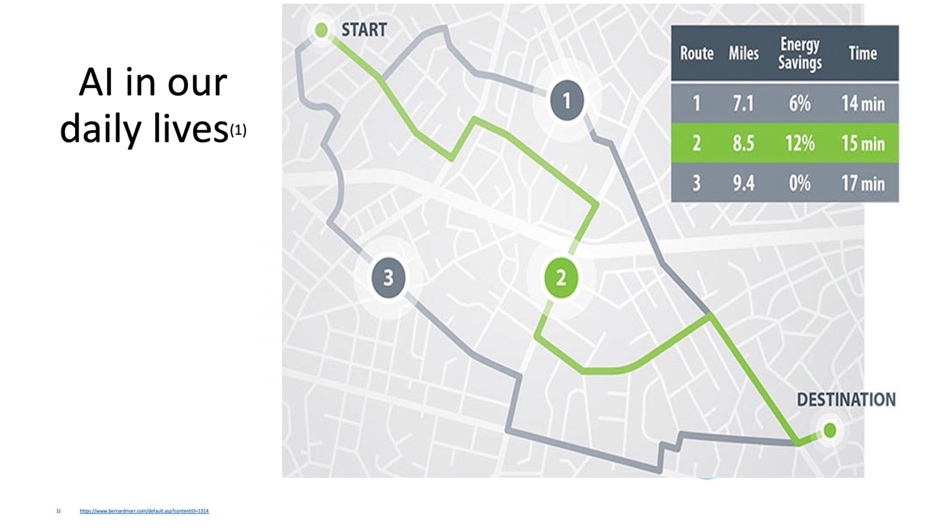


**Optimization and Scheduling:** While driving to work, people might use Google Maps, which uses AI to monitor live traffic conditions, compare them to previous traffic conditions, use weather information to recommend the best route to drive to the destination. In another collaborative work between Google and US National Renewable Energy Laboratory, Google is trying to find more echo-friendly routing in Google maps which allows users to co-optimize travel time and energy consumption for individual vehicles, fleets, or entire transportation networks. As you can see in the picture, the green route indicates the best energy saving level (12%) with average reasonable time (15 min) to get to the destination


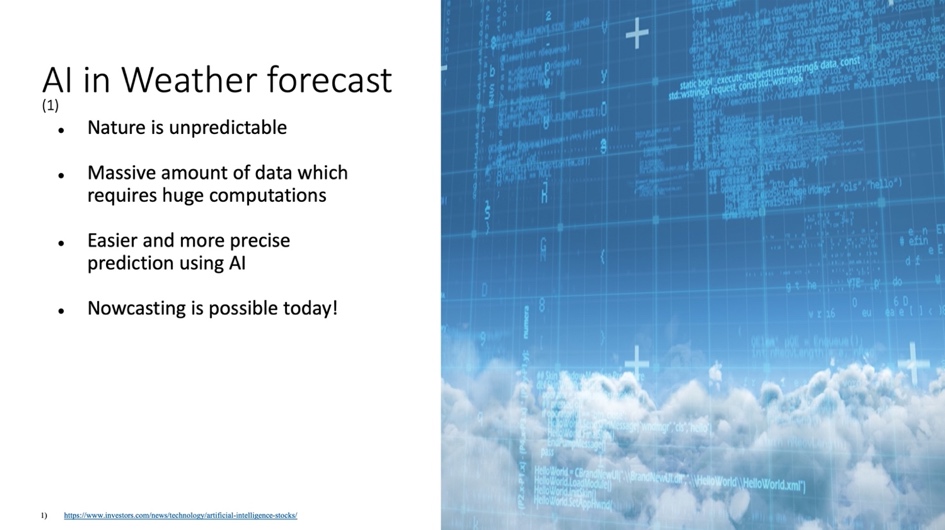


**AI in Weather forecast:** The chaotic nature of climate makes it almost impossible to make real-time predictions. For decades, weather forecast, in best scenario, had been 6 to 12 hours behind the real data. The good news is that now big companies such as Microsoft or Google are feeding all weather signals of the past from the satellites into Artificially Intelligent networks, memories to better model a weather prediction in advance. This have had huge impacts on agriculture businesses and supply chain management. Results also indicates that these computational models are cheaper, faster and more accurate compared to the more conventional approaches of weather forecasts. The weather forecasting and how AI is helping in forming a physics free understanding of weather is quite noticeable nowadays, helping to turn forecasting into nowcasting!


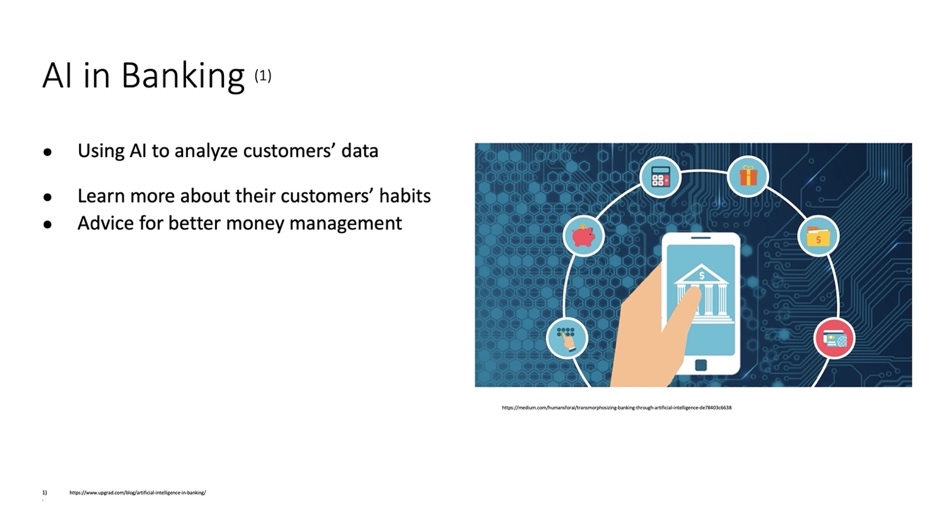


**AI in Banking:** AI has the potential to modify today’s banking. Large Canadian Banks such as TD, for instance, are using AI to analyse data which it uses to learn more about customers and anticipate their needs.


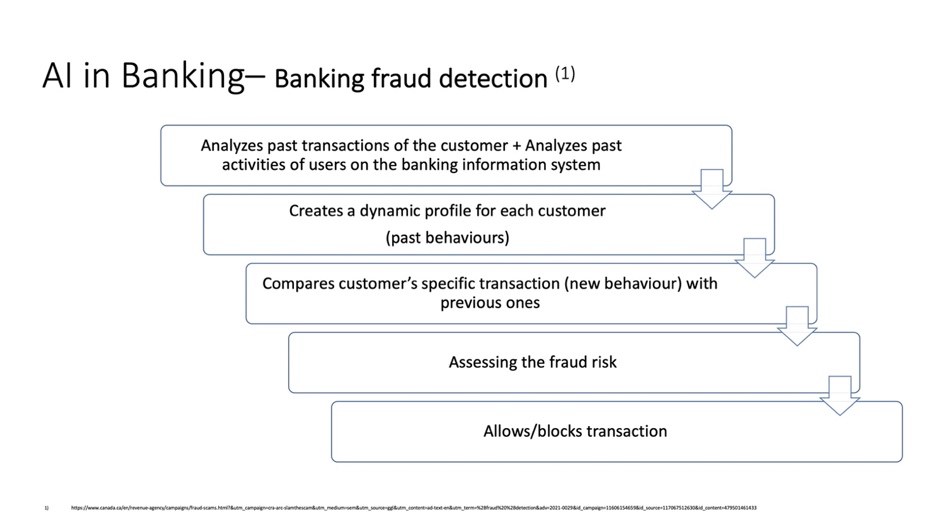


**Banking fraud detection:** When it comes to the Fraud detection, AI can be used to monitor financial transactions and user behaviour every time to detect suspicious activities or financial transactions. AI is capable of analysing the past transactions of the customer to learn their transactional behaviour as well as past activities of users on the banking information system and create a dynamic profile for each customer. Therefore, AI would be able to compare the customer’s specific transaction against the customer profile and compute a risk score out of it. If the risk score is sufficiently high, the AI machine will decide to block the transaction and qualify it for further investigation by the bank.

**Appendix B.**

Focus Group Discussion Interview Guide

**Introduction**

Hello. Welcome everyone. My name is Pooria Ghadiri. I am an MSc student at the department of Family Medicine, McGill University. I would like to start off by thanking each of you for taking time to participate today. We will be here for about 60 to 90 minutes. The purpose of this gathering is to gather your input on ‘Exploring the perceived needs of Primary Care Physicians (PCPs) about Artificial Intelligence (AI) systems in the care of adolescents’ mental health**.’**I’m going to lead our discussion today. I will be asking you questions and then encouraging and moderating our discussion. Dr, Mark Yaffe, professor in the Department of Family Medicine, McGill University and St. Mary's Hospital Center, is participating in this FG session predominantly as an observer, as well as to provide support for me should it be required. I will guide the conversation by asking questions that each of you can respond to. There are no right or wrong answers to these questions. We want you to share your thoughts with us so that we can get a broad range of ideas and opinions. If you wish, you can also respond to each other’s comments, hopefully in friendly, non-judgemental fashion, like you would in an ordinary conversation. It is my job to make sure that everyone here gets to participate and that we stay on track. I also would like you to know this focus group will be audio-visually recorded both through Zoom or WebEx software and an external digital audio recorder. The identities of all participants will remain confidential. The recording allows the research team to revisit our discussion for the purpose of consolidating the ideas and opinions expressed.

**Ground Rules**

To allow our conversation to flow more freely, I’d like to go over some ground rules:

1. First, before the focus group discussion begins, we want to ensure that all the members who agreed to participate today have received and signed off the consent form through an email.
2. We want you to do the talking. We hope everyone will participate according to their level of comfort.
3. Only one person speaks at a time. This is doubly important as our goal is to make a written transcript of our conversation today. It is difficult to capture everyone’s experience and perspective on our audio-visual recording if there are multiple voices at once.
4. There are no right or wrong answers. Every person’s opinions and experiences are important. Speak up whether you agree or disagree. We expect and want to hear a wide range of opinions and we do not anticipate consensus, just sharing.
5. Please avoid side conversations or virtual chatting through the Zoom or WebEx platform while the discussion is on.
6. We emphasize that what is said in this virtual room should remain here. You should be able to share anything if sensitive issues come up. Please do not disparage other participants’ remarks.
7. You may use each other names in the discussions, but I will not report your names or who said what. You may, however, later discuss with others what was addressed here, as long as you do not disclose who said what. Audio-visual recordings will be secured by the Principial Investigator, Dr. Samira Rahimi at McGill University’s One-Drive network. We may provide summary details of the study in oral or written reports once the study is complete.
8. I understand that I should keep both the audio and video on to ensure the discussion flow during the focus group. However, while I will plan to have no personal interruptions during the focus group, should that unexpectedly occur, I will click off the audio and video and re-open them as soon as possible afterwards.
9. In the end, I am happy to take questions if there are any.

**Introduction of the Participants**

May I ask if you could please introduce yourself and tell us a little about your practice. Let’s start from Dr …

**Demographic Questions**

Thank you so much for your attendance and sharing your valuable inputs. We would like to know a little about your demographic backgrounds. I will launch a very brief questionnaire through a link provided in a chat box. You should be able to see it running on your screen and be able to answer the question accordingly. Your answers will be anonymous and confidential. <https://forms.gle/4WTGp6HL6t6BvALfA>

Study title: Primary Care Physicians’ Perceptions of Artificial Intelligence systems in The Care of Adolescents’ Mental Health.

Please answer the following questions:

1. What is your gender? Female; Male; Non-binary
2. How many years have you been working in medical practice? (Please write down)
3. What percent of your patient care time is devoted to the care of adolescents in the ambulatory setting? (Please write down)

**FG Opening Question**

1. **Have you faced any challenges in the care of adolescents in the ambulatory setting?**

**Tips to initiate: Would anyone like to start the discussion to the following?**

- - Probing questions:
    - If yes, what are they?
    - Note: Now they have given me general responses. I need to break it down by asking: **“**Is the difficulty that you experience one that occurs only when you are treating a physical problem? Or when you are treating a mental health problem? Or is it all the time?”
    - More specifically, what about their mental healthcare---how do you approach it? Are you comfortable with it? What might make it better?  What might make it easier?

**Key Questions**

1. **Can you think of any way AI technology that could help in adolescents’ mental healthcare? (For example, in diagnosis, treatment, management or any other related fields?)**
   - Probing questions:
     - What made you think of that approach?
     - Does anyone agree or disagree with that suggestion? Why?
2. **What might be some of the possible benefits of using AI in adolescents' mental healthcare?**

Note: (Be ready to break down the question in the three probing aspects below)

- - Probing questions:
    - Benefits: to patients? to a care provider? To the healthcare system?

1. **What might be some of the possible risks or down-sides of using AI in adolescents' mental healthcare?**
2. **How comfortable would you feel in using AI in your practice?**

Note: This is a very broad question- Ask it first. If participants said they don’t really have a good answer for it, then I need to break it down:

- - Ok… I think I need to break down the question for you. Thinking purely about using “diagnostics,” how comfortable/uncomfortable would you feel in using AI ? And why?
  - I have to set reference this question based on the information I am getting from participants. Comfortability or comfortability can further be explained based on the area that a participant is talking. (I can ask the below areas later on if I hear nothing about later on)
  - Probing questions:
    - Impact on workload
    - Positive and negative reactions from patients, uncertainty about how to interpret the AI data,
    - Questions about how their colleagues might react to using AI (How your colleagues might react to using AI?)

1. **If you thought you'd like to try AI in your practice, what would be needed to give it a go? (What would you need to get started?)**
2. **Are there any financial implications (positive or negative) to you using AI in your practice?**
3. **Are you interested to be involved in the design and development stage of AI systems for your practice?** Break this question into two parts:
   - **If YES, who would be interested and why?**
   - **If NO, who would not be interested and why?**
4. **Do you think there is a need for more continuing professional development such as courses, seminars, workshops for doctors on the theme of AI as we have discussed it here today?** Break this question into two parts:

**Closing:** We have gotten to the end of our session. Thanks for coming today and talking about these issues. Your comments have given us lots of different ways to see this issue. But before doing so, are there any additional comments that people would like to make?

**Appendix C.** A *priori* codes

|  | Main codes and sub codes | Code Definition | Where to use it? | Where not to use it? | Examples in the transcript |
| --- | --- | --- | --- | --- | --- |
| 1 | **General challenges of using AI in adolescents’ care in ambulatory setting** | Primary care Physician’s (PCPs) particular challenges in adolescents’ medical services performed on an outpatient basis, without admission to a hospital or other facility. It is provided in settings such as: Offices of physicians, other health care professionals and hospital outpatient departments. | Any details about the PCPs past or present challenges regarding adolescents’ health care problems such as Injuries, violence, mental health, alcohol substance use, eating, mood disorder, early pregnancy, childbirth, etc. | Nothing outside ambulatory care | **Exp:** “*One of the main challenges with adolescence is the paucity of information that we get from them. It's hard to phrase, your questions in a way that will be open ended enough that they will actually provide some neat*.”  **Exp:** “*So, what can AI do to persuade the patient to do it or take the treatment? I don't know! Can AI do something about it? From our point of view, we can still talk to the patient tried to persuade the patient. And I think that if the patient has confidence in us, eventually some patients may agree to the treatment*”  **Exp:** “*So, I sort of see this as a personal challenge to connect, at least if you're going to be following someone for a mental health issue on the first one, two or maybe three visits, it's really just a relationship building. It's not really about therapeutics and diagnosis for me anyway when I'm when I'm looking at one of my patients who may be experiencing a mental health issue takes time to get to know them and figure out if we should be looking at any kind of therapeutics or not so that challenge of making a good connection.*” |
| 2 | **Use of AI in adolescents’ mental health care** | Refers to the possible and most likely potentials of using AI in adolescent mental health care to assist PCPs in providing more efficient care to adolescents such as using AI in diagnostics, screening, follow-ups, triage, etc. | Any details on PCPs perceptions on most potential AI tools that could assist them specifically with their services to the adolescents with mental health care from Intake, triage and screening to the management and follow-up. | Details on PCPs perceptions of using AI tools that could assist them with all sorts of patients’ population (not specified to the adolescents’ population) | **Exp: “***Yeah, I certainly think that it will be a lot easier because you can really a lot of questions, maybe it will be easier for the adolescents just to check off a box and say ‘do you take drugs’ and, you know, ‘have you been in prison?’, ‘Have you had, you know, then to say directly to the doctor?, and also I think the AI can work as, just as when we order something on Amazon, they know what you order and they know the next time around and you have ads about something that you bought. So, I think you can just pop up see for example if the, the young person checked off smoking then right away, the next question would be like cannabis or alcohol and related things so it's like a profiling of the of the of the patient. It does help, right?! to make us think of any other associated conditions that would be, you know, if the patients, and so ‘yes’ to one question, then it brings us to another ‘associated condition’ that we should not forget, or we might sometimes forget a question she might forget to ask.***”** |
| 3 | **Benefits of using AI in adolescent’s mental health care** | Refers to the possible advantages of using AI in adolescent’s mental health care at the level of patients (adolescents), PCPs and health care system. | Any details on possible benefits of using AI in adolescent’s mental health care at the level of patients (adolescents), PCPs and health care system such as understand how adolescents’ mental health problems develop, how it can be recognized more efficiently, how they spread, and how it can be treated, managed, prevented, etc. | We are looking for only the advantages of using AI at the level of patients (adolescents), PCPs and health care system. We are not looking into disadvantages or where if AI might harm adolescents’ mental health care | **Exp:** *“Sort of do that manually at the Herzl, we actually have like an intake or adolescent patient sort of referred, and then the second coordinator actually reviews, what exactly the referrals for and if it's something more urgent, we will be able to triage it. But again, this is very manual and that's the person behind it. But that's great that can be used as data, you know to train a system to do it automatically right?! you'd be able to reach more people and the human resources are the expensive things both in time and money and you know they're hard too hard to do, so I'd love to have somebody sitting in my second office here trashing my patients and spending an hour with them and getting distilling, a lot of, you know data for me. It's just not you know cost effective. I'd love to be able to do it, but I dream of having that and to have that as an AI bot or system. For me, that would be a really great help.”* |
| 4 | **Down-sides of using AI in adolescents’ mental health care** | Refers to the possible downsides of using AI in adolescent’s mental health care at the level of patients (adolescents), PCPs and health care system. | Any details on possible downsides of using AI in adolescent’s mental health care at the level of patients (adolescents), PCPs and health care system such as understand how adolescents’ mental health problems develop, how it can be recognized more efficiently, how they spread, and how it can be treated, managed, prevented, etc. | We are focusing solely on identifying the potential disadvantages of using AI at the level of patients (adolescents), PCPs, and the healthcare system. This includes concerns such as automation-spurred job loss, privacy violations, algorithmic bias, and other factors that could potentially harm any of these levels. We are not examining the advantages or where AI might benefit adolescents' mental health care. | **Exp:** *“When I went through ICU* [Hospital’s ICU] *12 years ago, I made the mistake of telling one of the docs there and I will tell you who it was. But one of the ICU docs* [working at the hospital’s ICU] *and I said: “how do you feel about the fact that in 2028 your job will be replaced by computer?”*  **Exp 2***:* “*I think it's important that the ‘human aspect’ of medicine is very important. We don't want the patient to feel that they've been treated by a robot. I don't think that I can show empathy towards the patients I think that's a very important part in in family medicine is that patient know us very well they feel empathy, even without doing anything just a fact of talking to a family doctor who listens to you. A lot of patients feel a lot better. I think that is the disadvantage. We don't want this to be like ‘robotic medicine’ for the patient.*” |
| 5 | **(PCPs’) Expected characteristic of AI tools (Yellow Font)**   - PCPs’ needs to begin using AI in adolescents’ mental health care - PCPs’ AI related continuing medical education (CME) - PCPs’ costs of/financial implications of using AI tool | Refers to all kinds of PCPs’ expected characteristics of AI tools that could potentially be used in adolescent’s mental health care services such as design features, the efficiency and ability of problem solving and decision making (performance expectation), perceived ease of use (effort expectation), AI tools’ maintenance, pre-requirements of using AI tools, value proposition of the AI tools to the PCPs, patients (adolescents) and health care system | Any details on PCPs’ expected characteristics of AI tools that could potentially be used in adolescent’s mental health care services from performance, design, structure, Incentives to use, financial implications, etc. | We are not looking for a single set of expected characteristics in PCPs | **Exp 1: “***I think you can still have a machine that has an output of answers for like let's say you don't want treatment A, and they go why? And I said, you know, you have the reasons that are common reasons patients can still select and you can give them the, you know, our explanation to why it actually might be a good idea or offer an alternative I think you can still get a computer do it.***”**  **Exp 2: “***For me, if it didn't require a lot of effort on my part and it did something useful for me, I guess I would give it a shot. You know, depends what kind of a tool we're talking about. I find a lot of computer type tools, look good on paper but then when you actually try to use them, it just takes an enormous amount of time and, you know, you have to, there's a learning curve, and you may not derive much benefit from them. So, it would have to be very easy to use, it would have to be seamless with my practice.***”**  **Exp 3: “**We *are flooded with so many courses and refreshers and I don't know how we can… umm.. I guess it's a new technology. I would be interested in doing it for sure, but hopefully we will spend the minimal time on this, and we can use it*.**”**  **Exp 4: “***also, the upfront cost is going to affect ‘which’ one we choose and ‘what’ we're going to use, just like with our EMR* [Electronic Medical Records] *like ‘cost’ did come out to be one of the factors. And if you're too expensive, we just don't look at you!****”*** |
| 6 | **PCPs’ involvement in design and development of AI tools** | PCPs’ determinant factors/decisive considerations for partnering in the manufacturing, design and development of AI tools in the care of adolescents’ mental health. | Any details on PCPs’ willingness/enthusiasm/terms of participation/perspectives on shouldering the responsibility to collaborate with AI tool designers, developers | Anything outside of definition | **Exp 1: “***It depends on what the tool is before. If it's something I think I will it in see my practice, then yes, I'd love to help out but if it's something I don't think might help my patients, I think we all have enough that we juggle!***”**  **Exp 2: “**If *we can get CME* [Continuing Medical Education] *credits for helping to develop some sort of AI solution, I mean this to me would be a dream come true! So, I'm all for that. There has to be an incentive of course I don't think we want to do this as a hobby, but we're definitely the best resource to tap when it comes to designing a system, I think often one of my biggest complaints with electronic medical record systems in general, is that a lot of them are built by engineers that don't have clinical experience right! So, we need to be tapped as a resource to develop these tools, but it has to be incentivized, either through credits or through something. There's got to be an ‘interest research’, or ‘even being paid for a time’, whatever that is, that would have to incentivize us from my in my opinion***”** |
| 7 | **PCPs’ medico legal aspects of using AI**   - **Need for AI governance regulatory bodies** | Refers to any process where any criminality is involved in PCPs’ working with an AI tool leading to mental and physical harm to the adolescents’ | Any details on PCP's perceived medico-legal responsibilities and professional obligations towards using AI in adolescents' mental health care to ensure the standard of care received. For instance, discussing appropriateness, quality, the functionality of the AI tools with patients, patient's preference, privacy risks, confidentiality and data protection, the robustness of data and reliability of the medical evidence informing the algorithm, applicable policies and guidelines (AI governance), etc. | Anything outside of definition | **Exp 1:** “*I think the AI is a great aid for us to help us make decisions. Just as we use, like ‘clinical guidelines’, but I think the ultimately the decision is a ‘human decision’ we have to make our decision. The guidelines don't tell us you must do this, and you must do that, and it helps! It guides us. I think, AI is the same thing, but we still ultimately are responsible for our decision and it’s a human decision!*”  **Exp 2:** “*Privacy’ is a big issue you have to separate the PII- the personally identifiable information- from the data, often that's a way to sort of keep things separate and safe but it's definitely a threat. All it takes is one breach. And, and people will lose, confidence as, as it happens with certain companies with our, you know, credit card information or personal information so imagine with health data and mental health on top of that, it can be disastrous.****”*** |
